# Focal cortical dysplasia lesion segmentation using multiscale transformer

**DOI:** 10.1186/s13244-024-01803-8

**Published:** 2024-09-12

**Authors:** Xiaodong Zhang, Yongquan Zhang, Changmiao Wang, Lin Li, Fengjun Zhu, Yang Sun, Tong Mo, Qingmao Hu, Jinping Xu, Dezhi Cao

**Affiliations:** 1 Shenzhen Children’s Hospital, Shenzhen, 518000 Guangdong China; 2 Shenzhen Institute of Advanced Technology, Chinese Academy of Sciences, Shenzhen, 518000 Guangdong China; 3 Zhejiang University of Finance and Economics, Hangzhou, 310000 Zhejiang China; 4 Shenzhen Research Institute of Big Data, Shenzhen, 518000 Guangdong China

**Keywords:** Drug-resistant epilepsy, Focal cortical dysplasia, Lesion segmentation, Transformer, Dual-self-attention

## Abstract

**Objectives:**

Accurate segmentation of focal cortical dysplasia (FCD) lesions from MR images plays an important role in surgical planning and decision but is still challenging for radiologists and clinicians. In this study, we introduce a novel transformer-based model, designed for the end-to-end segmentation of FCD lesions from multi-channel MR images.

**Methods:**

The core innovation of our proposed model is the integration of a convolutional neural network-based encoder-decoder structure with a multiscale transformer to augment the feature representation of lesions in the global field of view. Transformer pathways, composed of memory- and computation-efficient dual-self-attention modules, leverage feature maps from varying depths of the encoder to discern long-range interdependencies among feature positions and channels, thereby emphasizing areas and channels relevant to lesions. The proposed model was trained and evaluated on a public-open dataset including MR images of 85 patients using both subject-level and voxel-level metrics.

**Results:**

Experimental results indicate that our model offers superior performance both quantitatively and qualitatively. It successfully identified lesions in 82.4% of patients, with a low false-positive lesion cluster rate of 0.176 ± 0.381 per patient. Furthermore, the model achieved an average Dice coefficient of 0.410 ± 0.288, outperforming five established methods.

**Conclusion:**

Integration of the transformer could enhance the feature presentation and segmentation performance of FCD lesions. The proposed model has the potential to serve as a valuable assistive tool for physicians, enabling rapid and accurate identification of FCD lesions. The source code and pre-trained model weights are available at https://github.com/zhangxd0530/MS-DSA-NET.

**Critical relevance statement:**

This multiscale transformer-based model performs segmentation of focal cortical dysplasia lesions, aiming to help radiologists and clinicians make accurate and efficient preoperative evaluations of focal cortical dysplasia patients from MR images.

**Key Points:**

- The first transformer-based model was built to explore focal cortical dysplasia lesion segmentation.
- Integration of global and local features enhances the segmentation performance of lesions.
- A valuable benchmark for model development and comparative analyses was provided.

**Graphical Abstract:**

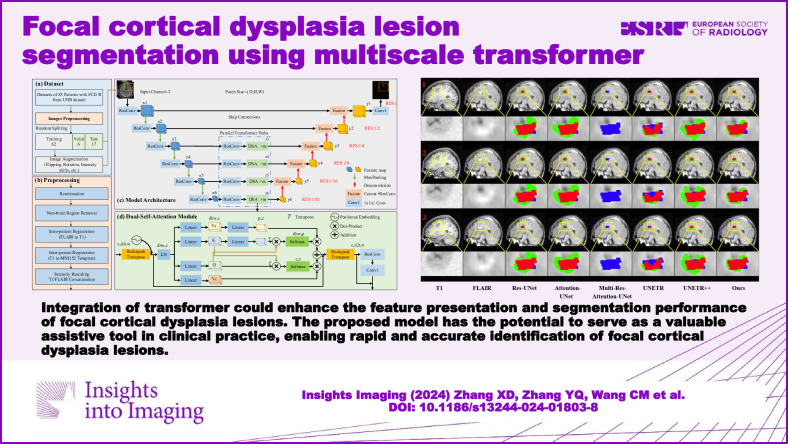

## Introduction

### Background

The most recent statistics suggest that approximately 70 million people worldwide are afflicted with epilepsy [1]. Focal cortical dysplasia (FCD) is a predominant cause of drug-resistant epilepsy (DRE) [2]. Surgical removal of FCD lesions is the most efficacious intervention for DRE-FCD, yielding seizure remission in about 70% of post-surgery patients [3]. The success of this intervention critically depends on the accurate localization and delineation of FCD lesions.

**Fig. 1:**
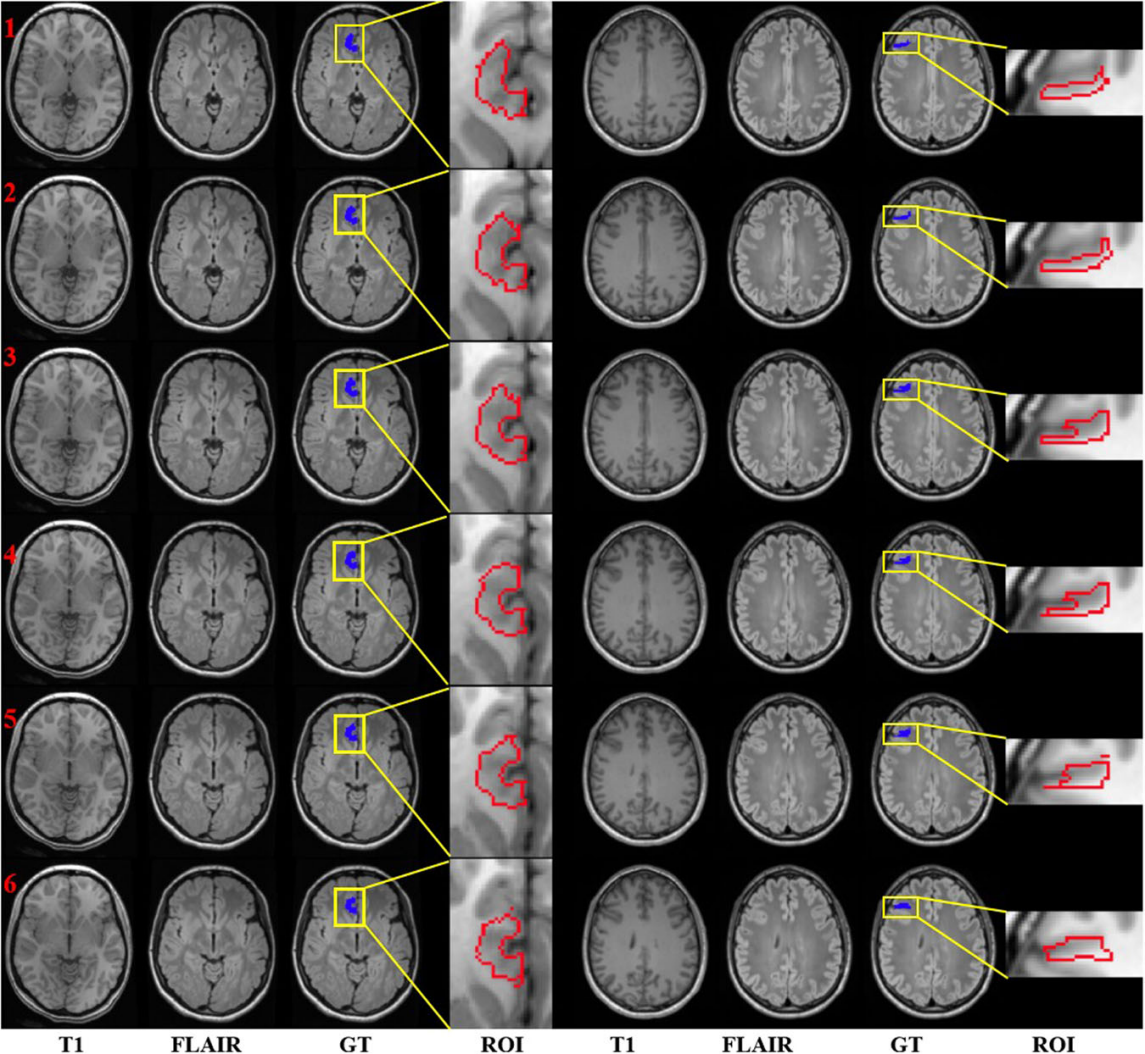
FCD lesion visualization on 6 consecutive MR slices of an MRI-positive patient in columns 1–4 and an MRI-negative one in columns 5–8. The lesion mask is rendered in blue. Region-of-interest (ROI) inside the yellow bounding box surrounding the lesion is enlarged on the right side, with the lesion contour drawn in red

However, identifying FCD lesions in clinical practice remains fraught with challenges. The structural changes associated with FCD on MR images can be subtle, and as such, are easily missed during routine visual inspections. Such changes include blurring of the gray-white matter junction (as shown in columns 1–4 of Fig. 1), cortical thickening, abnormal sulcal or gyral patterns, hyperintense signals on T2-weighted or fluid-attenuated inversion recovery (FLAIR) sequences, and the transmantle sign [4]. Furthermore, approximately one-third of FCD cases are classified as MRI-negative [5], as shown in columns 5–8 of Fig. 1, further complicating the clinician’s task to localize and evaluate FCD lesions effectively. In light of these challenges, there is an urgent demand for computer-aided techniques capable of detecting and quantifying FCD lesions in an automated and objective manner.

### Related work

Over the past few decades, the computer-assisted detection of FCD lesions has been pursued using voxel-based morphometry (VBM) [6] and surface-based morphometry (SBM) [7]. The VBM process involves registering images to standardize their spatial orientation and achieve voxel-wise alignment, followed by voxel-wise statistical analysis [8, 9]. Machine learning models can be trained for voxel-wise classification [10, 11]. However, VBM methods are sensitive to image registration and individual brain variances, with non-linear registration potentially altering local cortical structures and leading to detection failures.

In contrast, SBM methods rebuild the cortical surface to extract vertex-specific features, such as cortical thickness, curvature, sulcal depth, and “doughnut” features. Machine learning models are then trained on these vertex features for vertex-wise classification to identify abnormal vertex clusters [12–14]. Despite their effectiveness in detecting FCD, SBM methods are computationally demanding due to the requirement for cortical surface reconstruction, a process that can take several hours per patient. Both VBM and SBM are reliant on hand-crafted low-level features that may not be sufficiently discriminative to differentiate between FCD lesions and normal structures.

With advancements in parallel computing devices and theories of artificial intelligence, deep learning techniques, particularly convolutional neural networks (CNNs), have shown significant potential in medical applications [15]. In recent years, CNNs have been leveraged to detect FCD lesions from MR images [16]. The detection of FCD lesions can be conceptualized as a voxel-wise classification problem as in [17–19]. Fully convolutional networks, such as UNet [20] and its variants (e.g., Attention-UNet [21], Multi-Res-UNet [22]), can generate dense predictions in an efficient, end-to-end manner and have seen broad application in various medical segmentation tasks. The UNet was initially trained to detect FCD lesions using FLAIR slices [23], and it was subsequently extended to 3D Res-UNet with residual convolution blocks [24]. The utilization of more information provided within the 3D patches significantly improved performance. The Multi-Res-Attention-UNet enhances the UNet by incorporating MultiResUNet with attention gating [25], designed to extract salient features that represent complex FCD lesions.

### Challenges and solutions

While CNNs have proven adept at learning compelling feature representations, their local receptive fields limit their capacity to capture long-range dependencies. In contrast, Transformer models excel at modeling long-range dependencies [26]. The vision transformer (ViT) is the first Transformer model applied to image analysis [27] and has been adapted for medical image analysis, achieving state-of-the-art performance across various segmentation tasks [28].

However, Transformer-based models have yet to be extensively explored for FCD lesion segmentation, and challenges abound when applying these models. Specifically, FCD lesions vary in shape, location, and size, potentially curtailing performance improvements due to the single-scale patch splitting employed in current Transformer models on 3D medical images. Furthermore, these models involve extensive parameters and computations, leading to potential overfitting. In addition, existing models trained and validated on proprietary data may lead to pronounced performance disparities. To overcome these limitations, we propose a multiscale transformer-based model for FCD lesion segmentation based on a publicly open dataset.

## Methods

### Datasets

**Fig. 2:**
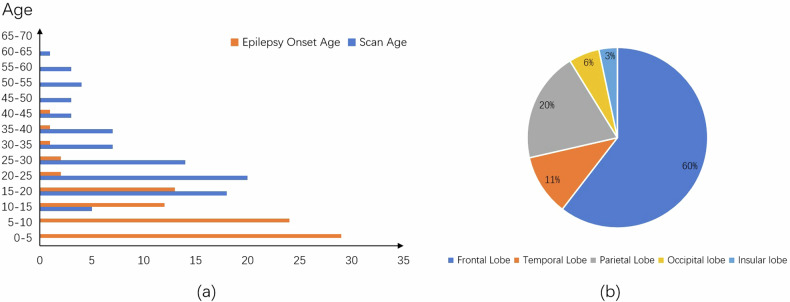
**a** Age distribution of the patients; **b** Lesion distribution in different brain lobes

A publicly open dataset, provided by the Department of Epileptology at the University Hospital Bonn (UHB dataset) [29], is used in this paper. Datasets of 85 patients diagnosed with FCD II, hospitalized from 2006 to 2021, were retrospectively collected, with approval from the university’s ethics committee. 50 of 85 patients (58.8%) are male. The scan age (28.9 ± 12.4 years) and epilepsy onset age (10 ± 8.3 years) distributions of the dataset are plotted in Fig. 2a.

Included in this dataset are MRI sequences for each patient, along with clinical information. MRI was performed using a 3 T MRI Scanner (Magnetom Trio, Simens Healthineers, Erlangen, Germany). T1-MPRAGE and fluid-attenuated inversion recovery (FLAIR) sequences were recorded.

Lesion ground truth was annotated on FLAIR images by two neurologists, based on a combination of diagnostic tools. A subset of 7 cases (8.3%) was categorized as MRI-negative or non-lesional. The lesions are located in the frontal lobe, temporal lobe, parietal lobe, occipital lobe, and insular lobe as shown in Fig. 2b.

**Fig. 3:**
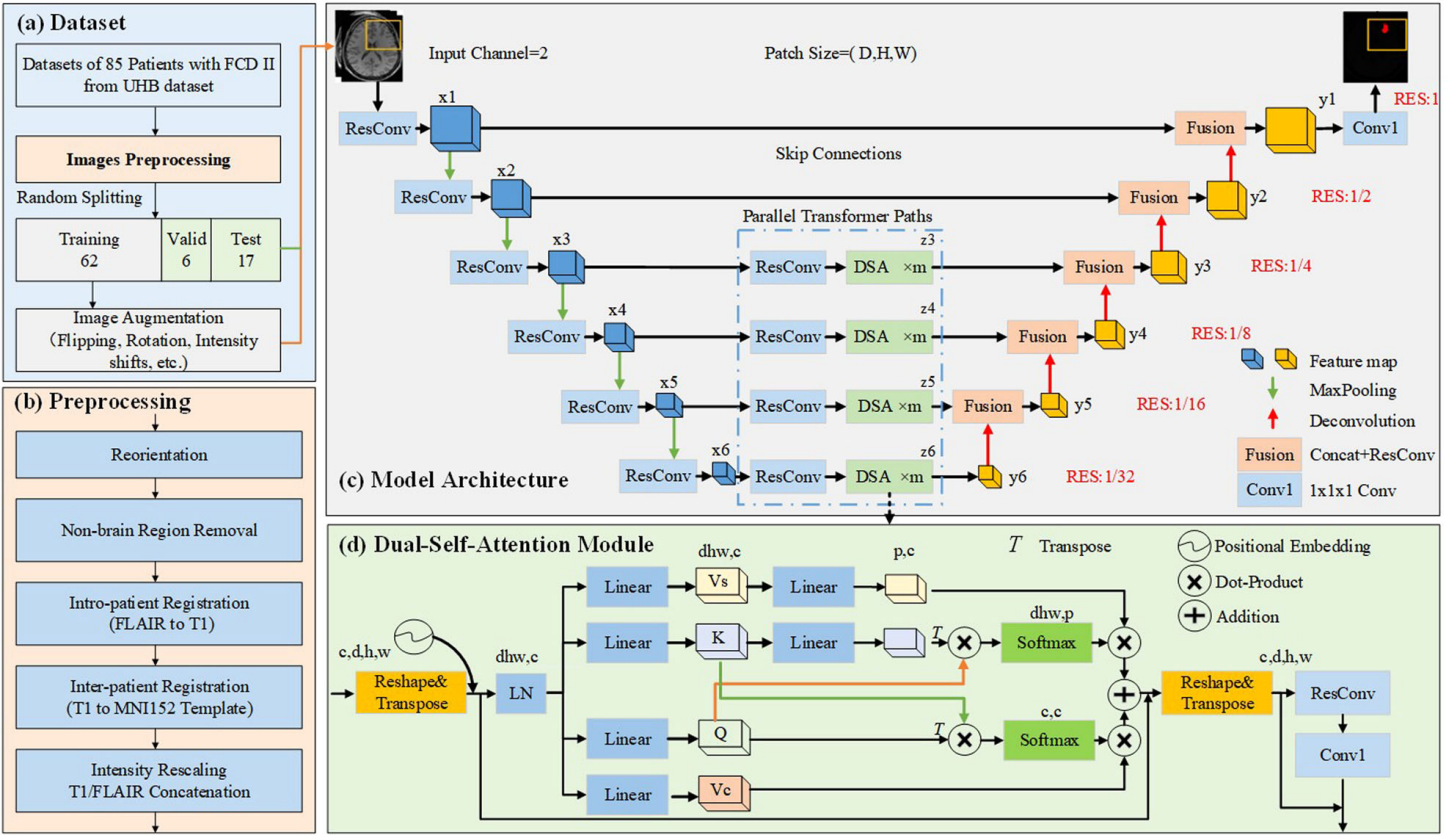
Flowchart of the proposed method. **a** Dataset splitting; **b** Preprocessing procedure; **c** Architecture of the proposed model based on an encoder-decoder structure. Parallel transformer pathways, each consisting of m dual-self-attention (DSA) modules, are inserted to capture the global features on feature maps of different resolutions, ranging from 1/4 to 1/32. **d** Architecture of the DSA module, consisting of a spatial self-attention branch and a channel self-attention branch

Figure 3 gives the flowchart of the proposed method. Preprocessing is firstly applied to align and normalize images as shown in Fig. 3a, b. Preprocessing details are introduced in Appendix 1. The dataset was randomly divided into a training set, a validation set, and a test set containing 62, 6, and 17 samples, respectively.

### Model architecture

The proposed multiscale dual-self-attention network (MS-DSA-NET) employs an encoder-decoder architecture with parallel transformer pathways connecting between them. The specific architectural details are elaborated below and represented in Fig. 3c. The proposed model accepts a 3D patch $x\in {R}^{{C}_{0}\times D\times H\times W}$ as input and produces a lesion probability map of the same size as the input, where$\,{C}_{0},{D},H,$ and ${W}$ represent the channels, depth, height, and width of the input patch, respectively.

The encoder within our proposed network is a six-level convolutional hierarchy. Each level incorporates a residual convolution module, referred to as$\,{ResConv}$, consisting of two convolutional blocks with residual connection. The feature representation at each level is mathematically expressed as:

1 $${x}_{i}\in {R}^{{2}^{i-1}\cdot C\times \frac{D}{{2}^{i-1}}\times \frac{H}{{2}^{i-1}}\times \frac{W}{{2}^{i-1}}},$$

where$\,i={{\mathrm{1,2}}},\cdots ,6$ corresponds to the layer index and *C* denotes the number of filters in the first convolutional layer and is fixed to 16 in our configuration.

### Transformer pathway

Our approach capitalizes on CNN feature maps at various resolutions to establish long-range relationships. Initially, the CNN feature map at each level undergoes processing through a ${ResConv}$ module to halve the feature channels and normalize the output. The normalized features are denoted as $x\in {R}^{c\times d\times h\times w}$, where *c* represents the feature channels and $d\times h\times w$ corresponds to the feature spatial size.

In contrast to using patch embedding as in ViT, the feature maps denoted as ${\{{x}_{i}\in {R}^{{c}_{i}\times {d}_{i}\times {h}_{i}\times {w}_{i}}\}}_{i={{\mathrm{3,4,5,6}}}}$ are directly fed into parallel transformer pathways. Each pathway comprises $m$ DSA modules. In our configuration, we have opted to set $m$ equal to 3.

#### DSA architecture

The detailed structure of the DSA module is depicted in Fig. 3d. We reshape and transpose feature map into a token sequence denoted as $x\in {R}^{n\times c}$, where $n={dhw}$ is the sequence length. A learnable positional embedding $e\in {R}^{n\times c}$, is added to the feature sequence to encapsulate the position information.

Subsequently, it undergoes normalization by a ${LayerNorm}$ layer. This is followed by two parallel self-attention modules that independently enrich spatial and channel features. Linear layers first map the feature sequence into four matrices. These represent the query $Q\in {R}^{n\times c}$, the key $K\in {R}^{n\times c}$, the spatial value ${V}_{s}\in {R}^{n\times c}$, and the channel value ${{V}}_{c}\in {R}^{n\times c}$. Both ${V}_{s}$ and ${{V}}_{c}$ are employed in their respective self-attention modules. Meanwhile, $Q$ and *K* are shared in both modules to avoid additional complexity.

#### Spatial self-attention (SSA) module

SSA is capable of emphasizing the salient regions of FCD lesions by capturing the global inter-position dependencies. To decrease the complexity of the conventional self-attention mechanism, linear layers are utilized in SSA to project *K* and ${V}_{s}$ into a lower-dimensional space:

2 $$\begin{array}{c}\bar{K}={Linear}\left(K\right)\in {R}^{p\times c},\\ \bar{{V}_{s}}={Linear}({V}_{s})\in {R}^{p\times c},\end{array}$$

where the projection dimension ${p}\ll n$. The spatial similarity matrix is then computed by multiplying the query matrix $Q\in {R}^{n\times c}$ by the transpose of the projected key matrix $\bar{K}\in {R}^{p\times c}$, followed by rescaling and ${Softmax}$ for normalization:

3 $${A}_{s}={Softmax}\left(\frac{Q{\bar{K}}^{T}}{\sqrt{s}}\right)\in {R}^{n\times p},$$

where the rescaling factor $s=c/{n}_{h}$ and ${n}_{h}$ is the number of self-attention heads. A spatial attention map is obtained:

4 $${x}_{s}={A}_{s}\bar{{V}_{s}}\,\in {R}^{n\times c}$$

With the aid of linear projection, the computation complexity of SSA is reduced to $O({np})$. This makes it feasible to apply parallel SSA on feature maps at multiple resolutions, ranging from $1/4$ to $1/32$.

#### CSA module

CSA is designed to focus more acutely on FCD lesion-related features by capturing the global inter-channel dependencies. CSA utilizes the shared $Q\in {R}^{n\times c}$ and the key $K\in {R}^{n\times c}$ with SSA to compute the normalized channel similarity matrix:

5 $${A}_{c}={Softmax}\left(\frac{{Q}^{T}K}{\sqrt{s}}\right)\in {R}^{c\times c},$$

where the rescaling factor $s=c/{n}_{h}$. Subsequently, the channel attention map is obtained:

6 $${x}_{c}={{V}_{c}A}_{c}\in {R}^{n\times c}$$

The spatial attention map ${x}_{s}$ and channel attention map ${x}_{c}$ are fused by addition. The input feature map $x$ is connected to the fused attention map via a residual connection:

7 $${z}^{{\prime} }=x+({x}_{s}+{x}_{c})$$

We reshape and transpose ${z}^{{\prime} }{\in R}^{n\times c}$ to ${z}^{{\prime} }\in {R}^{c\times d\times h\times w}$, with the aim of recovering the spatial information and making it suitable for the following convolutional module. The convolutional module consists of a ${ResConv}$ module and a $1\times 1\times 1$ convolution with a residual connection to generate the enriched feature maps.

### Decoder architecture

The decoder’s function is to fuse features from various hierarchical levels, an operation facilitated by deconvolution and specialized Fusion blocks. A Fusion block receives encoder features, denoted as either ${z}_{i}$ or ${x}_{i}$, to fuse feature maps ${y}_{i}$ from a deeper level as inputs, thereby enabling concatenation and convolution. The feature maps, ${y}_{i}$, are initially upsampled via deconvolution using a kernel and stride of $2\times 2\times 2$. This process synchronizes feature maps across different resolutions and simultaneously reduces the number of feature channels by half prior to entering the Fusion block. Ultimately, the feature maps are refined through a $1\times 1\times 1$ convolution. This effectively transforms the feature channels into two output channels, which is immediately followed by a *Softmax* layer to yield a normalized probability map.

### Loss functions

The optimization of the proposed model employs a hybrid loss function as the objective function, which comprises two distinct components: a regional term and a voxel term:

8 $$\begin{array}{c}L={L}_{{dc}}+\omega * {L}_{{ce}},\\ {L}_{{dc}}=1-\frac{2 \, * \, {\sum}_{i}{P}_{i}{G}_{i}+\varepsilon }{{\sum}_{i}{P}_{i}+{\sum}_{i}{G}_{i}+\varepsilon },\\ {L}_{{ce}}=-\left({\sum }_{i}{G}_{i}\log {P}_{i}+(1-{G}_{i})\log ({1-P}_{i})\right),\end{array}$$

where reginal term ${L}_{{dc}}$ is the Dice loss to evaluate the disparity between the predicted lesion map, and the ground truth; voxel term ${L}_{{ce}}$ is the cross-entropy loss to evaluate the dissimilarity between the predicted probabilities and the ground truth labels at the voxel level; $P$ is the prediction map and $G$ is ground truth; $\omega$ is a predefined weight, which in this study is set to 1.

### Evaluation metrics

We employed two primary metrics: subject-level and voxel-level assessments. At the subject level, we leveraged detection sensitivity (${sSens}$) and the average number of false-positive clusters (${nFPC}$). An FCD lesion was considered detected if there was at least one-voxel overlap between the prediction and the ground truth, a criterion consistent with that used in [19]. $\,{sSens}$ is defined as follows:

9 $${sSens}=\frac{{TPs}}{{TPs}+{FNs}},$$

where ${TPs}$ denotes true positive subjects, and $F{Ns}$ represents false negative subjects.

${nFPC}$ quantifies the count of false-positive lesion detections within FCD patients. Clusters are initially delineated based on voxel connectivity analysis, with any cluster lacking actual lesion voxels being considered a false-positive cluster.

At the voxel level, we used the commonly applied metrics of precision (${Prec}$), sensitivity (${Sens}$), and the Dice coefficient (${DC}$) for evaluation. These metrics are defined as:

10 $$\begin{array}{c}{Prec}=\frac{{TP}}{{TP}+{FP}},\\ {Sens}=\frac{{TP}}{{TP}+{FN}},\\ {DC}=\frac{2\times {TP}}{2\times {TP}+{FN}+{FP}},\end{array}$$

where ${TP}$ refers to the number of true positive voxels, ${FP}$ indicates the number of false-positive voxels, and ${FN}$ denotes the false negative voxels.

## Results

To validate the performance of our proposed method, a series of experiments were conducted. Various current state-of-the-art CNN models were implemented for comparison, including Attention-UNet [21], Res-UNet [24], and Multi-Res-Attention-UNet [25], which have previously been employed for FCD lesion segmentation. Additionally, transformer-based models, specifically UNETR [30] and an extended version, UNETR + + [31], were included in the comparative analysis. Training details are provided in Appendix 2.

### Comparisons among different models

#### Quantitative comparisons

**Fig. 4:**
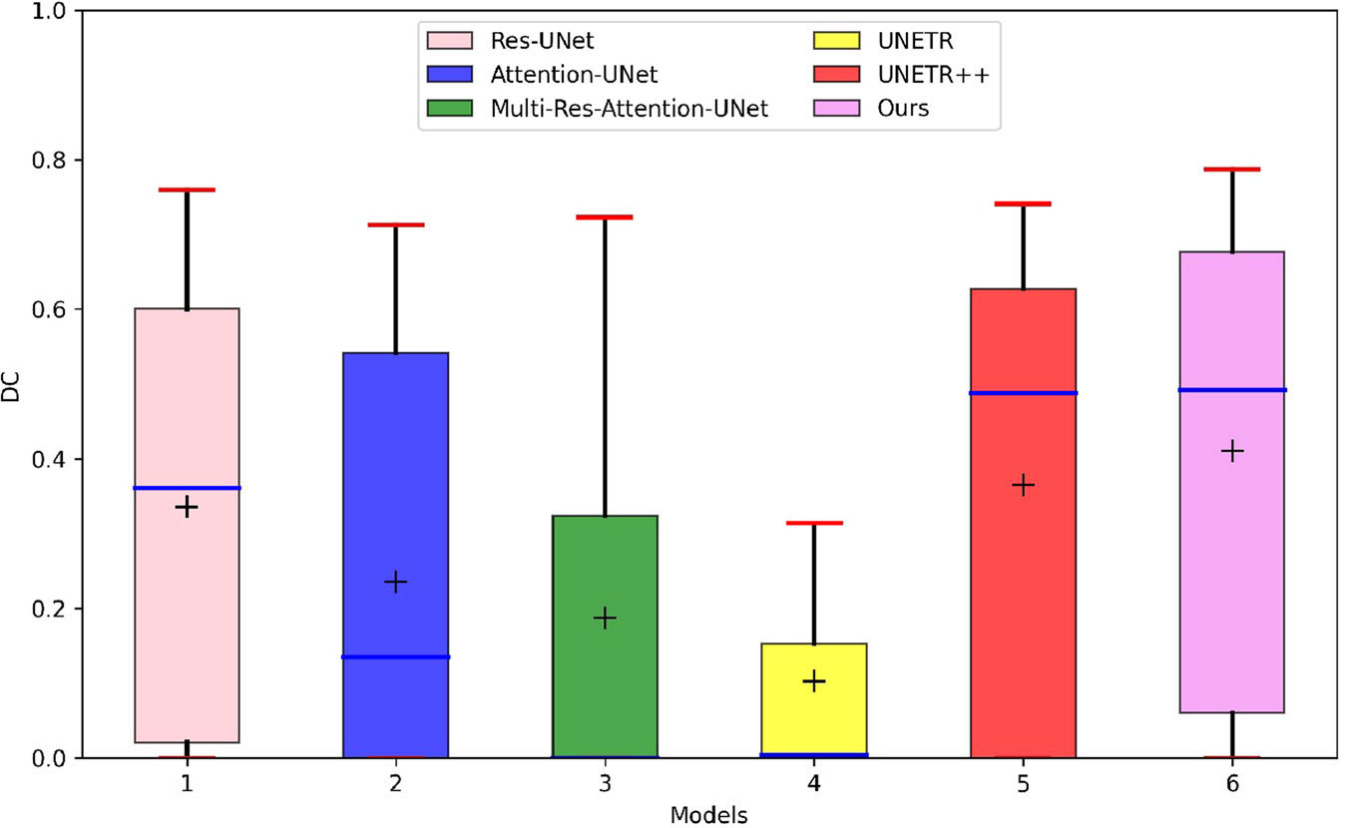
Dice coefficient (DC) comparisons among different models. “+” represents the position of mean DC; the Blue line marks the position of median DC

Subject-level and voxel-level evaluations were conducted for each patient in the test set. To quantify ${nFPC}$, connected component analysis was employed on the segmentation masks to identify and categorize separate clusters. Any cluster that did not overlap with the ground truth was classified as an ${FPC}$. The outcomes of these evaluations for FCD lesion segmentation can be found in Table 1. We also counted the number of ground truth lesions, true positive lesions, false-positive lesions, and false negative lesions as listed in Table 2. In addition, Fig. 4 provides the box-plot of DC values among different models for intuitive performance comparison.

**Table 1:** Quantitative comparisons with state-of-the-art methods using subject-level and voxel-level metrics

*Methods*	*sSens*	*nFPC*	*Sens*	*Prec*	*DC*
Attention-UNet [21]	0.647	1.118 ± 1.906	0.246 ± 0.258	0.336 ± 0.359	0.236 ± 0.268
Res-UNet [24]	0.765	1.235 ± 1.214	0.372 ± 0.295	0.385 ± 0.334	0.335 ± 0.265
Multi-Res-Attention-UNet [25]	0.471	3.000 ± 3.597	0.228 ± 0.314	0.219 ± 0.307	0.187 ± 0.262
UNETR [30]	0.588	1.529 ± 2.304	0.115 ± 0.171	0.344 ± 0.399	0.115 ± 0.171
UNETR + + [31]	0.706	0.882 ± 0.900	0.436 ± 0.340	0.382 ± 0.321	0.373 ± 0.295
**MS-DSA-NET (ours)**	**0.824**	**0.176** ± **0.381**	**0.438** ± **0.324**	**0.481** ± **0.343**	**0.410** ± **0.288**

${sSens}$ denotes the sensitivity at the subject level, while ${nFPC}$ refers to the average number of false-positive clusters identified. Additionally, ${Sens}$ and ${Prec}$ represent the sensitivity and precision at the voxel level, respectively. Lastly, ${DC}$ signifies the Dice coefficient score, which is a statistical measure of similarity between two sets of data

The bold values provide an intuitive way for readers to find the best performance value of each metric

**Table 2:** Comparisons of the number of ground truth lesions (GT lesions), true positive lesions (TP lesions), false-positive lesions (FP lesions), and false negative lesions (FN lesions) among different models

*Methods*	*TP lesions*	*FP lesions*	*FN lesions*	*GT lesions*
Attention-UNet [21]	11	19	6	17
Res-UNet [24]	13	21	4	
Multi-Res-Attention-UNet [25]	8	51	9	
UNETR [30]	10	26	7	
UNETR + + [31]	12	15	5	
**MS-DSA-NET (ours)**	**14**	**3**	**3**	

The bold values provide an intuitive way for readers to find the best performance value of each metric

#### Qualitative comparisons

**Fig. 5:**
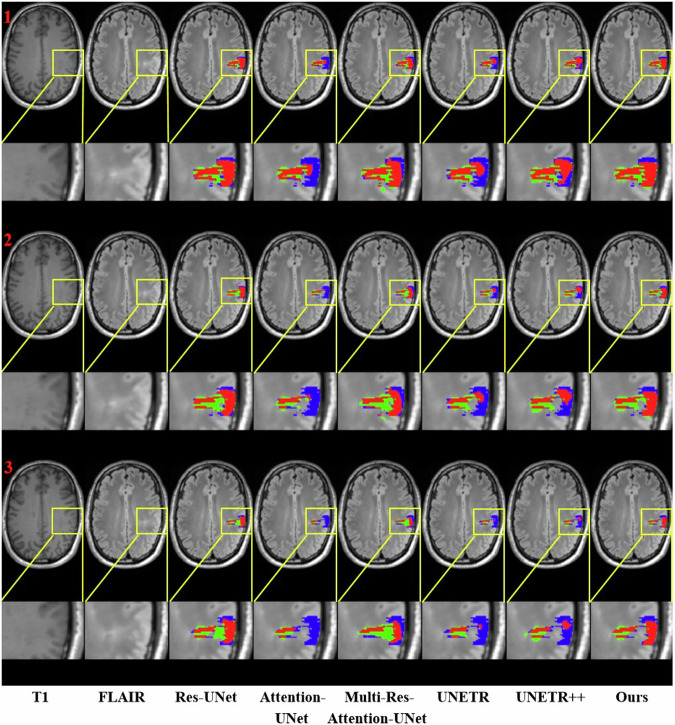
Visualization of segmentation results of one typical case. Consecutive 3 axial slices along with segmentation results of different models are drawn in rows. Red, blue, and green colors represent the true positive (TP), false negative (FN), and false positive (FP), respectively. ROIs inside the yellow bounding box encapsulating the lesions are drawn below

For a qualitative comparison of FCD lesion segmentation, segmentation results of one representative case are visualized in Fig. 5. It provides three consecutive axial slices overlapped with prediction maps of different models. The ROI encapsulating lesion and prediction map are enlarged for a better view. The proposed model achieved a DC of 0.787, higher than other models.

### Ablation studies

Experiments were conducted to explore the influence of model architecture on performance. Initially, acknowledging that T1 and FLAIR imaging sequences provide distinct insights into the structural alterations linked to FCD lesions, experiments were designed to assess the performance of models trained with varying input sequences. The evaluation outcomes are elaborated in Table 3.

**Table 3:** Ablation experiment results on different input sequences using the proposed method

*MRI sequences*	*sSens*	*nFPC*	*Sens*	*Prec*	*DC*
T1 only	0.588	1.000 ± 1.138	0.286 ± 0.311	0.339 ± 0.336	0.262 ± 0.254
FLAIR only	0.588	0.706 ± 1.225	0.325 ± 0.324	0.302 ± 0.321	0.299 ± 0.305
**T1&FLAIR**	**0.824**	**0.176** ± **0.381**	**0.438** ± **0.324**	**0.481** ± **0.343**	**0.410** ± **0.288**

The bold values provide an intuitive way for readers to find the best performance value of each metric

We conducted a series of experiments to validate the improved performance provided by different self-attention mechanisms. In our study, we replaced the DSA module in the proposed model with the SSA module and CSA module, respectively, and carried out experiments under the same configuration. The comparative performance of models with different self-attention modules is illustrated in Table 4.

**Table 4:** Ablation experiment results on different self-attention mechanisms

Self-attention modules	*sSens*	*nFPC*	*Sens*	*Prec*	*DC*
SSA	**0.824**	0.765 ± 0.876	**0.482** ± **0.301**	0.458 ± 0.324	**0.419** ± **0.267**
CSA	0.765	0.824 ± 0.785	0.441 ± 0.337	0.328 ± 0.298	0.328 ± 0.281
**DSA**	**0.824**	**0.176** ± **0.381**	0.438 ± 0.324	**0.481** ± **0.343**	0.410 ± 0.288

Specifically, within the MS-DSA-NET architecture, the original dual-self-attention (DSA) module was substituted with two alternative self-attention modules: the spatial-self-attention (SSA) module and the channel-self-attention (CSA) module

The bold values provide an intuitive way for readers to find the best performance value of each metric

The size of the patch is essential for transformer-based models to extract meaningful semantic features. To evaluate the influence of patch size on segmentation performance, we conducted experiments using MS-DSA-NET with various patch dimensions. Within our experimental setup, we set the patch sizes to be $64\times 64\times 64$, $96\times 96\times 96$, and $128\times 128\times 128$, respectively. The performance comparisons among these different patch sizes are outlined in Table 5.

**Table 5:** Ablation experiment results of MS-DSA-NET with different patch sizes

Patch size	*sSens*	*nFPC*	*Sens*	*Prec*	*DC*
64^3^	0.765	5.588 ± 2.724	0.459 ± 0.309	0.306 ± 0.265	0.342 ± 0.254
96^3^	0.765	2.294 ± 1.707	0.445 ± 0.328	0.339 ± 0.279	0.352 ± 0.257
**128**^**3**^	**0.824**	**0.176** ± **0.381**	**0.438** ± **0.324**	**0.481** ± **0.343**	**0.410** ± **0.288**

The bold values provide an intuitive way for readers to find the best performance value of each metric

## Discussion

This study introduces a novel multiscale transformer-based network for the segmentation of FCD lesions. Experiments have been conducted to validate its performance on the UHB dataset. Our results illustrate the improved performance achieved by integrating multiscale DSA modules. To the best of our knowledge, this is the first study applying a transformer-based model for the segmentation of FCD lesions.

Our experiments demonstrate that the proposed MS-DSA-NET outperforms both CNN-based and transformer-based models. As shown in Table 1, MS-DSA-NET achieves the highest subject-level sensitivity of 0.824, successfully identifying lesions that overlap with the ground truth mask in 13 out of 17 patients, while maintaining a relatively low average number of false positives per case (${nFPC}$ = 0.176 ± 0.381).

At the voxel level, the proposed model performs 9.9% and 3.7% better in terms of ${Prec}$ and $D$*C* compared to the second highest-ranked model (UNETR + +), respectively. The UNETR, based on a traditional ViT architecture, demonstrated the poorest performance among all models, potentially due to its extensive parameter count, leading to overfitting and reduced generalization when trained on limited datasets.

The proposed method offers a flexible framework for combining information from different sequences or imaging modalities, potentially enhancing segmentation task performance. An ablation study on input sequences reveals that the proposed model’s performance using multi-channel input (T1 + FLAIR) is superior to that using T1 or FLAIR alone, both at the subject and voxel levels. The fusion of T1 and FLAIR improved the detection rate from 0.588 to 0.824 and reduced ${nFPC}$ from 1.000 (T1) and 0.706 (FLAIR) to 0.176. Additionally, it achieved improved voxel-level $D$*C*, ${Sens}$, and ${Prec}$ scores of 0.410 ± 0.288, 0.438 ± 0.324, and 0.481 ± 0.343, respectively, surpassing single-modality input performances. Future work will explore the incorporation of PET imaging, a crucial modality for detecting subtle FCD lesions, to further enhance MRI-negative sample segmentation.

Further ablation studies on different attention mechanisms validated the effectiveness of the DSA block. The SSA model achieved the same detection rate as the DSA model (0.824); however, the ${nFPC}$ for the SSA model increased to 0.765 ± 0.876, compared to 0.176 ± 0.381 for the DSA model. The complementarity of the CSA module to the SSA module was also assessed. While it contributed to recognizing false-positive classifications, it slightly decreased the voxel-level $D$*C* metric. The study also found that using patches of size $128\times 128\times 128$ achieved the best performance at both the subject and voxel levels, indicating that larger patches provide more global information beneficial for enhancing FCD lesion feature representation and improving false-positive discrimination.

**Fig. 6:**
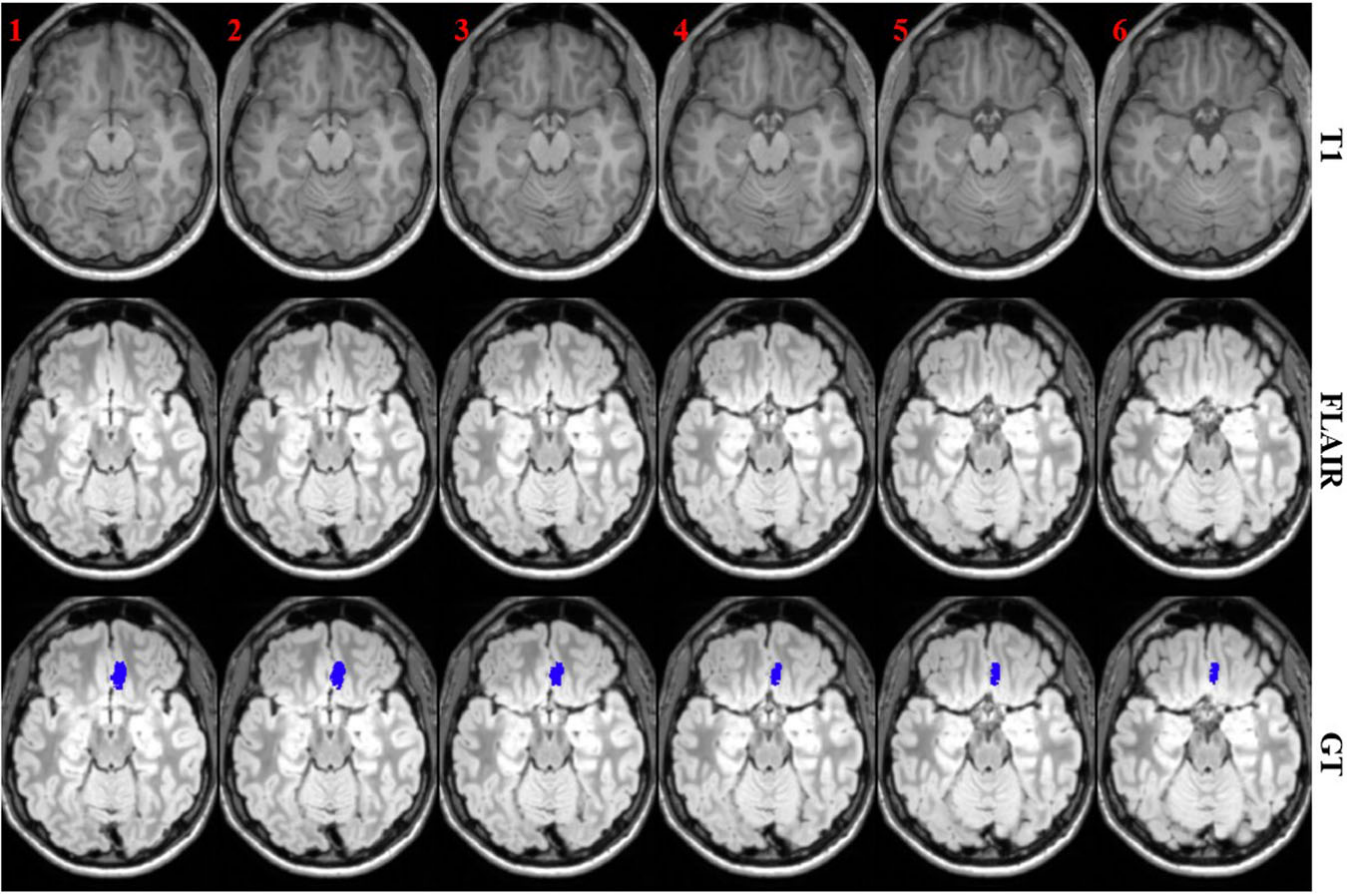
One case that failed to detect any lesion. The lesion ground truths are superimposed on the FLAIR slices in blue, highlighting the regions of interest

Limitations of our proposed model include the inability to identify lesions in 3 patients, resulting in an overall detection sensitivity of 82.4%. MRI slices of one failed case are depicted in Fig. 6. The lesion area masked in blue is poorly defined and represents a challenge for detection. Furthermore, the $D$*C* value for FCD lesion segmentation is 0.410, which is low compared to other medical segmentation tasks, highlighting the inherent challenges of FCD lesion segmentation. While numerous studies have investigated this area, they have typically relied on private datasets, leading to discrepancies in data volume and performance metrics. A fair comparison across common datasets would be beneficial. Given the difficulty in collecting FCD datasets with expert annotations, and considering that no other studies on this public UHB dataset have been reported, our method could serve as a valuable benchmark for future model development and comparative analyses in FCD lesion segmentation. In addition, this paper focuses on lesion detection of FCD patients. We will include normal controls to further evaluate the model performance in our future work. An auxiliary task to discriminate between FCDs and normals is worth trying to include in the model, aiming to further enhance performance.

## Conclusion

This study introduces a novel method for segmenting FCD lesions from multi-channel MR images using a multiscale transformer-based network. Experimental results substantiate the exceptional performance of the proposed model in the segmentation of FCD lesions. The method outlined in this paper holds promise as a valuable tool for medical practitioners, empowering them to swiftly and accurately detect FCD lesions.

## Supplementary information

ELECTRONIC SUPPLEMENTARY MATERIAL

## Data Availability

The public UHB dataset used in this paper is available at https://openneuro.org/datasets/ds004199/.

## Abbreviations

CNNs: Convolutional neural networks
CSA: Channel self-attention
DC: Dice coefficient
DRE: Drug-resistant epilepsy
DSA: Dual-self-attention
FCD: Focal cortical dysplasia
MS-DSA-NET: Multiscale dual-self-attention network
nFPC: Number of false-positive clusters
Prec: Precision
SBM: Surface-based morphometry
Sens: Sensitivity
SSA: Spatial self-attention
sSens: Subject-level sensitivity
VBM: Voxel-based morphometry
ViT: Vision transformer
