# Supplementary material for: Focal cortical dysplasia lesion segmentation using multiscale transformer
Source: Insights Imaging. 2024 Sep 12;15:222. doi: 10.1186/s13244-024-01803-8 (PMC11393231; doi:10.1186/s13244-024-01803-8)
Supplement: Supplementary file 1 — ELECTRONIC SUPPLEMENTARY MATERIAL [file 13244_2024_1803_MOESM1_ESM.pdf]

Focal Cortical Dysplasia Lesion Segmentation Using Multiscale Transformer

ELECTRONIC SUPPLEMENTARY MATERIAL

Appendix 1. Dataset Preprocessing

MRI preprocessing was conducted using FSL6.0.7 for both T1 and FLAIR images. The T1 images were initially re-oriented to a standard direction using *fslreorient2std*, then auto-cropped to remove non-brain regions with *robustfov*, the transformation of which is denoted as S_{norm}. Following this, intra-patient rigid registration was performed to align FLAIR images with their corresponding T1 images, denoted as S_{intra}. An inter-patient affine registration was subsequently applied to align the T1 images with the 1mm isotropic *MNI152* brain template, resulting in a transformation represented as S_{inter}. With these transformation matrices, we obtained the aligned images:

$$\begin{gathered} t1 = S_{inter}(S_{norm}(T1)), \\ flair = S_{inter}(S_{norm}(S_{intra}(FLAIR))), \\ g = S_{inter}(S_{norm}(S_{intra}(GT))). \end{gathered} \tag{1}$$

We then truncated the intensity of all voxels in the preprocessed volumes T1 and FLAIR to the percentage range of *[0.5%,99.5%]* to reduce extraneous details and enhance image contrast, followed by intensity scaling to *[0,1]*. The normalized T1 and FLAIR were concatenated into a 2-channel volume.

Appendix 2. Training setup

The model was developed using PyTorch version 1.12 and MONAI version 1.2.0, and the coding was done in Python 3.8. The experimental setup comprised a graphics workstation that ran a 64-bit Ubuntu 18.04 operating system. The workstation was powered by an Intel(R) Xeon(R) Silver 4116 CPU operating at 2.10GHz, equipped with 94 GB of RAM, and supported by 2 Nvidia Tesla P100 GPUs, each offering 16GB of memory.

During the training phase, we adopted a strategy of random sampling where patches centered on lesion (positive) and non-lesion (background, negative) voxels were extracted in a balanced 1:1 ratio. Each patch was uniformly sized at $128 \times 128 \times 128$ voxels. These image patches were batch-processed by the proposed model to generate lesion probability maps. These maps were subsequently employed to compute the loss value in comparison with the ground truth using the loss function. In the DSA module, the projection size p was set to 64.

For optimization purposes, we selected the AdamW optimizer for its capability to minimize the loss value and facilitate model parameter updating, with an initial learning rate of 1e-4. The model was trained over the course of 1000 epochs, with a batch size of 4. We implemented a validation process on the validation set at intervals of every 20 training epochs to mitigate the risk of over-fitting, and an early-stopping strategy was employed based on the validation results. We provide a test script with pretrained model weights at https://github.com/zhangxd0530/MS-DSA-NET. It could be used as an assistant tool to

help clinicians find the FCD lesion candidates for further treatment, such as stereoelectroencephalography or surgery resection.

Further, to enhance data variability and bolster model robustness, we incorporated data augmentation techniques during the training process. The augmentation transforms applied included random flips in the axial, sagittal, and coronal planes; random rotations around the axial axis within an angular range of *[-30, 30]* degrees; random intensity shifts; and the injection of random Gaussian noise.
